# Understanding Tourists’ Perceptions of Animal Welfare, Governance, and Conservation: Evidence from the Panda Base

**DOI:** 10.3390/ani15243548

**Published:** 2025-12-10

**Authors:** David Fennell, Yulei Guo, Richard Butler

**Affiliations:** 1 Department of Geography and Tourism Studies, Brock University, St. Catharines, ON L2S 3A1, Canada; dfennell@brocku.ca; 2 Chengdu Research Base of Giant Panda Breeding, Chengdu 510081, China; yulei.guo@panda.org.cn; 3 Strathclyde Business School, University of Strathclyde, Glasgow G1 1XQ, UK

**Keywords:** governance, conservation, welfare, visitor perception, Panda Base, wildlife tourist attraction

## Abstract

Wildlife viewing has long been a central feature of tourism. However, growing concerns over the welfare of animals involved have raised critical questions about human management and the use of wildlife. In response, scholars have developed frameworks to assess and guide the operations of animal-based attractions. This paper applies such a framework to the Chengdu Research Base of Giant Panda Breeding in China, empirically examining visitor perceptions of animal welfare management practices. Drawing on survey data from over 1000 visitors, the study finds that, overall, tourists hold positive evaluations of the Base’s governance, welfare, and conservation practices. However, these perceptions are influenced by factors such as prior exposure to panda-related social media and individual knowledge of animal welfare, highlighting the role of mediated experiences in shaping visitor judgments. The findings underscore the need for further research on ethical wildlife tourism practices and suggest that engaging and empowering tourists—through education, participatory evaluation, and digital platforms—can enhance both public awareness and the effectiveness of conservation and welfare initiatives.

## Simple Summary

This study examined tourist perceptions of governance, conservation, and animal welfare at the Chengdu Research Base of Giant Panda Breeding using governance-welfare-conservation indicators. Such insights are critical given the ethical challenges posed by growing public interest in wildlife-based tourism. Based on over 1000 survey responses, visitors generally rated the Base’s management practices positively, particularly in restricting visitor contact with animals and promoting conservation benefits. Social media experience was found to influence perceptions, with more engaged visitors providing higher evaluations. Despite overall positive views, the findings highlight the need to further enhance visitors’ knowledge and awareness of key welfare and conservation indicators to support ethical wildlife tourism practices.

## 1. Introduction

Animals have been important to humans since prehistory, as sources of food, aides in hunting, transportation, protection, and, in more recent centuries, for companionship and amusement. In the last role, they have been viewed as attractions, as features of entertainment and fascination, particularly in the context of tourism. In that role, they have long been used for transportation, particularly in relatively inaccessible locations, as novelty forms of carriage, and especially as attractions to be viewed, both in the wild and in captivity. One animal in particular is the Giant Panda, whose international appeal is reflected in its adoption as the insignia of the World Wildlife Fund, its use as an agent in political relations (“Panda diplomacy”), and, in the process, it has become a major attraction in zoos and wildlife reserves worldwide. Despite this widespread global awareness, they remain endangered, and governments that house Giant Pandas guard their animals diligently, most of them on loan under a commercial arrangement with the government of China. The species is widely respected in its native China. Sites such as the one reported here are key to attempts to re-establish the species more widely in the wild, and they have become significant tourist attractions in their own right.

In 2023, Polaris Market Research [1] estimated that wildlife tourism, encompassing activities such as ecotourism, hunting, fishing, captive animals, and animal-riding, will generate approximately US$303 billion by 2032, at an annual growth rate of 6.9%. Giant Panda tourism belongs to and contributes to this expanding global market. Indeed, it has been argued [2] that wildlife tourism accounts for approximately 20–40% of all international tourism, reflecting its economic value.

The ways animals are used and displayed across varied geographical contexts have raised significant conservation and welfare concerns, reflecting a growing conviction that the instrumental treatment of animals in tourism is increasingly seen as ethically unacceptable [3]. In their comprehensive study on animal-based tourism, Moorhouse et al. [2] found that most wildlife tourism attractions (WTAs) had negative conservation and animal welfare impacts on the animals used, reflecting the absence of sound standards and policies. Subsequently, Moorhouse et al. [4] found that primed respondents (those asked introductory questions about likely conservation and animal welfare impacts on WTA venues) were more likely to prefer wildlife tourism venues with beneficial operations than those with detrimental operations. Based on these findings, the authors concluded that visitors need more accurate information about the conservation and welfare standards of such sites in order to make better-informed decisions about whether to visit them. Fennell’s [5] study on animal welfare literacy extended the work of Moorhouse et al. [4] by focusing on the need to understand tourists’ levels of animal welfare literacy. He identified five categories: awareness, knowledge, attitudes, skills, and action, as being important in recording any move from animal welfare illiteracy to literacy in the context of animal-based tourism, supporting previous studies calling for enhanced conservation [6] and animal welfare [7] learning as being valuable for the benefit of animals and the tourism industry. While this framework highlights the role of visitors in making informed and ethical choices, it also implies that wildlife tourism operators share responsibility in fostering such literacy by providing accurate information and educational opportunities. Tourists’ recognition, attitudes, and knowledge regarding WATs and their associated animal welfare standards constitute critical determinants of WATs’ market performance [8,9,10,11]. This aligns with previous studies that call for enhanced conservation and animal welfare education to benefit both animals and the tourism industry.

The importance of protecting the wellbeing of animals used in tourism led to the development of frameworks to assess conservation and animal welfare, as well as the proposal of more ethical attractions for animal-based operations [12,13]. More comprehensive frameworks have recently been advanced by Meyer et al. [14] and Fennell et al. [15] to categorize standards of welfare and conservation, enabling more sustainable animal-based attractions and better educating tourists about the impacts of animal use. Tourists’ perceptions of animal welfare practices within WAT venues provide a foundational basis for subsequent educational initiatives. To date, however, there has been an absence of studies that empirically test these frameworks in specific settings involving targeted species in the view of tourists, which this study helps to fill. The purpose of this study is to use the Fennell et al. [15] prototype framework to investigate how tourists visiting the Chengdu Research Base of Giant Panda Breeding (Panda Base) respond to questions on the governance, conservation, and welfare of animals housed at the Base. This study answers the following two research questions:

1. How do tourists perceive the implementation of animal welfare, governance, and conservation practices at the Panda Base?
2. What factors shape tourists’ recognition of animal welfare, governance, and conservation at Panda Base?

The paper is organized as follows: First, animal welfare, governance, and conservation—identified by Fennell et al. [15] as three key performance criteria of WATs—are introduced, along with the corresponding measurements that frame this study. Second, the data collection process at the Panda Base and the statistical methods employed are described. Third, the collected data are presented and interpreted. Finally, the paper discusses the implications of the findings and offers suggestions derived from the study.

## 2. Literature Review

### 2.1. Conservation, Animal Welfare, and Governance

Biodiversity encompasses the totality of genes, species, and ecosystems [16], while biodiversity conservation centres on how well or poorly we are conserving these natural entities [17]. Conservation concerns have traditionally focused on habitat loss, invasive species, pollution, human population growth, and overharvesting, and, more recently, on the impacts of climate change. New research indicates that nature-based tourism should also be included in the list of human-induced rapid environmental change (HIREC) agents alongside historically recognised forms [18]. Research has revealed biodiversity conservation challenges resulting from tourism, including prey vulnerability [19], habituation [20], impacts from roads and trails, and vegetation trampling and trail erosion [21]. Despite this evidence, nature-based tourism has long been regarded as a conduit through which money spent at animal-based attractions is believed to support the conservation of both charismatic and less charismatic species [22,23].

The welfare of animals used in tourism had traditionally been a concern only to the extent that it reflected their ability to sustain the viability of tourism operations [23,24]. This mindset is changing as travel companies like Intrepid [25] and Responsible Travel Responsible [26,27] make statements about the need for a tourism industry that protects animal welfare. Non-profit organisations, such as Volunteer Forever, have also embraced animal welfare discourses in arguing that “Sadly, not all animal interactions are what they seem” [27], noting that travellers are unaware of the impacts that they have on the animals they covet, echoing the work of Moorhouse et al. [2], above. Animal welfare has been defined as an individual’s “state as regards its attempts to cope with its environment” [28] p. 524. Broom [29,30] further extends the concept to refer to physical, environmental, behavioral, and psychological aspects of animals. The confluence of science, activism, and political will to improve the quality and quantity of care provided to animals in their use is being emphasized [31].

Including the concepts (and practices) of governance in NEWT reinforces Rutherford’s [32] ideas on power and control in our management of animals in tourism. This power and control is manifested through networks of knowledge and practice and shapes certain truths on how we view nature, including the fact that animals are rarely considered in the rational processes and systems that underpin governance structures, and also through the commodification of nature as agents and organizations shape animals and their ecosystems according to material costs and benefits according to gender, race, and class. The global ecotourism industry, Rutherford claims, allows us to still feel good about ourselves even though it is still based on the commodification of nature for some at the expense of others [32].

How we regulate nature falls in the domain of biopolitics, as the “management of life” [32] p. xiii. In major tourist destinations, e.g., Disney’s Animal Kingdom, corporate biopolitics works through fantasy, in which concepts like conservation become household words because of the magnitude of Disney’s economic, political, and cultural power. Conservation is elevated as an idea in theme parks such as Disney’s, often masking consumption, especially the discourse of conservation as spectacle [33]. Rutherford argues that “Disney commodifies our vision and understanding, and through that commodification, governs what we come to know as nature” (p. 88). In animal-based tourism studies, governance and power have been discussed in the context of local practices that have come into conflict with formidable forces, namely global principles and standards set by organizations with their own interests in mind [34,35].

While the animals in wildlife tourism venues have been rendered “*affectless*” in the absence of “capacity, agency, and force” through regulation 193 in [32,36], these attractions also govern the conduct of tourists, using a range of tools and techniques including codes of ethics and certification schemes [15] and even self-regulation in the case of the International Association of Antarctica Tour Operators [37]. While governmental and industry stakeholders argue that their conservation and welfare activities are sound, evidence is to the contrary. Sheppard and Fennell [38] found little regard for animal welfare and conservation in policy documents, and Font et al. [39] found industry lacking in enforcement of animal welfare claims despite the implementation of various standards. A study by Jones and Comfort [40] on seven tourism companies with animal welfare statements on their websites identified four main themes: (1) a commitment to animal welfare, (2) policies on human–animal contact, along with policies on various animal and animal groups, (3) the importance of the role of different stakeholder groups involved with these companies related to the welfare of animals, and (4) how such measures ought to be monitored and audited. The authors acknowledge the cultural and geographical reach of these companies and the real potential for these companies to fail to meet their policies and commitments to animal welfare due to cultural differences.

### 2.2. Impact Assessment Frameworks

The need to more comprehensively assess the conservation and welfare impacts on wildlife tourism species has led to the development of several frameworks. Rodger et al. [13] developed an assessment framework for the sustainable development of whale shark operations in Australia and for marine wildlife in general. Ecological and environmental criteria were developed (e.g., threatened status, life cycles of species, known behaviour, feeding behaviour, habitat), as well as operational and social criteria (e.g., type of activity, accessibility, licenses required by operators, frequency of interaction, interpretation, and monitoring of interactions with focal species). Conservation issues emerged as important, but animal welfare is a minor aspect of their framework, as is the case with other frameworks. Higginbottom et al. [12] suggest that “Although some wildlife tourism activities probably make a net positive contribution to conservation…there is a large body of evidence demonstrating that tourism based on free-ranging wildlife can often lead to negative consequences for those animals…” (pp. 1–2). Their framework is more managerial in nature, focusing on stakeholder input, management objectives, management actions, monitoring programs, leading to an assessment of whether or not objectives are being met (e.g., visitor numbers, spatial and temporal distribution, design of experience, involvement in conservation, environmental damage, and so on). Overall, most studies on tourism with focal species in marine environments typically focus more on conservation issues [41,42] than on animal welfare.

This trend has begun to change, and Senigaglia et al. [24] write that, because tourists are seeking close encounters with wildlife [43], they are often caught in a complex web of justifying these encounters in the face of potential negative impacts. In a study by Naylor & Parsons [44] on public attitudes and perceptions of cetaceans, the authors found a general lack of awareness of the conservation status of these species, underscoring the need for greater outreach to inform the public. In general, tourists are unaware of the impacts they have on wildlife in many contexts [4].

Meyer et al. [14] developed a semiquantitative assessment model for the sustainability of wildlife tourism operations consisting of five factors: tractability (management and community support), socio-economic values, conservation outcomes, animal welfare, and impacts on ecosystems. Another recent model by Fennell, Moorhouse, and Macdonald [15] focuses on governance, conservation, and animal welfare, with 27 standards embedded across these three domains. This latter framework advances tourist literacy [2,45] in the absence of WTA operators’ motivation to improve animal welfare and conservation standards, by allowing tourists to assess WTAs’ ethical standards. Appendix A is a summary of the standards, organised into three categories, based on a comprehensive literature review and drawing on case studies of lions in African WTAs and tiger WTAs in Thailand. The authors conclude that “what is urgently required is a robust set of criteria by which non-expert tourists can externally assess—and accurately represent to others—standards at WTAs” [15] p. 22.

## 3. Method

### 3.1. Research Site

The Chengdu Research Base of Giant Panda Breeding (Panda Base), located near the city center of Chengdu, is the world’s largest ex situ Giant Panda conservation facility. According to its official website, approximately 230 Giant Pandas reside at the site. Prior to the COVID-19 pandemic, visitor numbers grew from 1.89 million in 2014 to 9 million in 2019. Panda Base has been a pioneer in wildlife scientific education in China and has received numerous national and provincial awards for its outstanding contributions to public wildlife conservation. In 2020, the UN World Tourism Organization recognized Panda Base in its report *Sustainable Development of Wildlife Tourism in Asia and the Pacific*, noting that the facility represents “an interim step towards the establishment of protected areas harboring wild sustainable populations” and highlighting its progress “in the breeding, release, and survival of pandas in the wild.”

In recent years, Panda Base has attracted increasing attention from tourism scholars [46,47,48]. Cong et al. [46] identify Panda Base as a key case for understanding wildlife tourism, noting that it ranks first among 126 attractions in Chengdu on TripAdvisor and draws both domestic and international visitors due to its large captive panda population. In a study evaluating the welfare of giant pandas at Panda Base, Fennell and Guo [48] report that visitors generally perceive the facility as performing well across most welfare indicators and in the implementation of informed consent.

### 3.2. Questionnaire Design, Collection, and Statistical Method

Following the framework of Fennell et al. [49], the authors developed a questionnaire to examine tourists’ perceptions of the Panda Base’s practices in animal welfare, governance, and conservation. Fennell et al., in 2 in [49], proposed that tourists’ perceptions of the three criteria in captive wildlife tourism attractions empower tourists “to make ethical choices of WTAs” by equipping them with tools to assess the “venues’ ethical standards”. Their framework also provides specific measurements for each criterion, which were adapted into multiple-choice items in the questionnaire.

The second author, working at Panda Base, translated the questionnaire into Chinese, paying attention to the specific cultural contexts of local and Chinese cultures, and using layperson’s language to increase its readability. The second author also translated the Chinese version back into English, which was reviewed by the first author to ensure the texts had retained their original meanings. An example of the original texts and translation is as follows (Table 1):

The questionnaire began with an informed consent statement, which all participants were required to acknowledge. In accordance with the *Measures for Ethical Review of Life Sciences and Medical Research Involving Human Subjects* (关于印发涉及人的生命科学和医学研究伦理审查办法的通知) issued by the Chinese Ministry of Education and the Ministry of Science and Technology, ethical review and approval may be waived for anonymous studies such as this. The consent form emphasized the voluntary nature of participation and clarified that respondents were free to withdraw from the survey at any time.

Nevertheless, the research strictly adhered to general ethical principles for studies involving human participants. The consent form emphasized the voluntary nature of participation, assured respondents of the anonymity of their responses, and informed them that they could withdraw at any time without consequence. All data were stored securely and used solely for academic research purposes.

Second, a demographic section was included, comprising nine questions on gender [50,51], age [50,52], marital status [53], education [52], place of residence [52], occupation [54], visitation experiences [50,55], social media engagement [56], and interests in the Giant Panda [48]. Following this section, the three criteria and their sub-questions were listed.

The third section contained the three criteria—governance, welfare, and conservation—with subset questions (governance: 9; conservation: 4; welfare: 12) (see Appendix A).

For all questions in Table 1, values were assigned to choices: Choice a = 1, Choice b = 2, Choice c = 3, and Choice d = 4. To reduce the recognition burden of tourists, all answers were structured so that Choice a indicated the best performance and Choice d the worst.

The questionnaire was administered through Wenjuanxing, an online survey platform that generated a QR code for participant access. The researcher printed the QR code and displayed it at a trolley stall near the south exit of the Panda Base, where data were collected on 9–10 December 2024. Interested visitors could approach the stall, scan the code with their mobile phones, and complete the survey. The second author was present to address participants’ inquiries and distribute small amenities, which facilitated engagement. This method has previously proven effective in attracting large, stable numbers of participants in field settings and has been successfully employed by the second author in several studies.

As a token of appreciation, participants received a Giant Panda magazine upon completing the survey. Wenjuanxing automatically ensured that all responses were submitted in full and converted the data into downloadable files for subsequent analysis.

A total of 1104 questionnaires were collected. One questionnaire was excluded because the respondent indicated “Disagree” on the consent form, and 11 questionnaires were removed due to implausible age entries (e.g., under 10 or over 100 years). This left 1092 valid responses for further analysis, which were saved in an SPSS (.sav) file on the second author’s working computer. Statistical analyses were conducted using SPSS version 26.0.0.2.

To address the research questions, a series of statistical analyses were conducted. Descriptive statistics were employed to examine participant characteristics and to provide an overview of tourists’ perceptions of governance, welfare, and conservation at the Panda Base. Additionally, variance analyses (*t*-tests and Kruskal–Wallis tests) were performed to assess the effects of demographic characteristics and visitation experiences on visitors’ perceptions.

## 4. Results

### 4.1. Demographic Profile and Visitation Summary

Table 2 presents an overview of participants’ demographic characteristics and visitation experiences at the Panda Base. The composition of the sample is consistent with previous surveys conducted at the site [48].

Table 3 indicates that tourists rated Panda Base’s governance, conservation, and welfare highly, with mean values across the three dimensions concentrated between 1.4 and 1.6 (close to the best option, Choice a = 1). Skewness and kurtosis values further suggest that responses were strongly clustered toward Choice a, reflecting a broad consensus among visitors that the Base performed well in these areas. The only item showing greater variability was the allocation of income to conservation (II4), which had a higher mean and variance, suggesting some uncertainty about the Base’s financial transparency. Within Governance, Question I6 (pursuit of best practice in training employees in welfare and conservation; M = 1.66) received the lowest rating, though the difference compared to the highest-rated item, I4 (truth in marketing; M = 1.53), was minimal. In Conservation, the strongest performance was observed in II3 (conservation benefits of captivity; M = 1.40). For Welfare, the highest rating was given to III9 (restrictions on visitor–animal contact; M = 1.38), while slightly lower evaluations were noted for III10 (animal reinforcement; M = 1.64).

### 4.2. Variation Tests for Demographic Differences and Visiting Experiences

To examine how different demographic groups varied in their perceptions of the Panda Base’s governance, conservation, and welfare performance, *t*-tests and Kruskal–Wallis tests (with prior normality checks) were employed. These tests compared demographic features across two groups (e.g., gender, social media interests) and more than two groups (e.g., age, education). Only demographic characteristics showing significant differences (*p* < 0.05) are reported.

Table 4 reveals that demographic characteristics with two categories also influenced tourists’ evaluations of the Panda Base. Marital status was one such factor: single or divorced participants tended to assign higher ratings across governance, conservation, and welfare items (e.g., I2, I4, I7, I8, III2, and III6) compared to married respondents. However, singles rated the Base lower on II4 (stated income allocation; M = 2.06) than married participants (M = 1.82), suggesting greater skepticism regarding financial transparency. Gender differences were minimal, with significant variation observed only for III6 (signs of obesity or malnutrition; *p* = 0.027) and III9 (touch policy; *p* = 0.002). Finally, first-time visitors generally evaluated the Panda Base more favorably than repeat visitors, indicating that prior exposure may moderate perceptions.

No significant differences were observed across age groups (<25, 25–30, 31–40, 41–50, >50) according to the Kruskal–Wallis test. Normality checks using the Shapiro–Wilk and Kolmogorov–Smirnov tests indicated that all variables had *p*-values below 0.001, justifying the use of non-parametric tests. Table 5 further shows that educational background significantly influenced evaluations, particularly regarding welfare items. While educational attainment did not generate notable differences in governance and conservation ratings, variation was more pronounced in welfare assessments. Overall, respondents with a bachelor’s degree tended to evaluate the Panda Base more favorably than those with other educational levels, except in II4 (stated income), where their ratings were lower.

A key finding was that participants’ engagement in Giant Panda-related social media significantly influenced their evaluations across all three dimensions (all *p* < 0.05) (Table 6). On average, participants who followed panda-related social media content rated the Panda Base more positively than those who did not. Standard deviations above 1.00 for non-followers indicate greater variability within this group, with many respondents more likely to select Choice d (“I am unaware of evidence of this criterion at Panda Base”). The largest discrepancy between groups emerged in item II4 (allocation of income to conservation; difference = 0.47). While both groups expressed some uncertainty (SD = 1.243 for followers, SD = 1.391 for non-followers), social media followers gave more favourable evaluations (M = 1.82) than non-followers (M = 2.29). The smallest difference was observed for I8 (volunteer programs at Panda Base; difference = 0.21). Social media followers rated III9 (touching policy; M = 1.31) as the best-performing item, while non-followers rated III6 (signs of obesity or malnutrition; M = 1.57) most positively. Despite these differences, both followers (M = 1.82) and non-followers (M = 2.29) considered II4 (stated income allocation) to be among the least transparent aspects of the Base’s practices.

These findings highlight that informational exposure and awareness played a central role in shaping tourists’ evaluations of the Panda Base. Social media engagement emerged as the most consistent predictor of favourable ratings, suggesting that followers of panda-related content arrive with greater awareness and more positive predispositions. Similarly, higher educational attainment, particularly at the bachelor’s level, was associated with more favourable assessments, though both groups flagged financial transparency (II4) as a persistent concern. Marital status and visitation history also moderated perceptions: single/divorced and first-time visitors tended to be more supportive, whereas married and repeat visitors tended to offer more cautious evaluations. In contrast, age and gender were largely non-influential, with only minor differences detected in specific welfare items. Taken together, the results indicate that communication channels (e.g., social media) and prior familiarity shape perceptions more than immutable demographic traits, underscoring the importance of transparency and targeted outreach in enhancing the Base’s public credibility.

## 5. Discussion

This study examined visitors’ perceptions of governance, conservation, and animal welfare at the Panda Base and found that visitors had an overall positive experience with the organization. The findings indicate that demographic characteristics such as gender, age, education, and marital status exert only a limited influence on visitors’ evaluations, suggesting that recognition of Panda Base’s role is relatively consistent across groups. One notable exception was the influence of social media: participants with prior exposure to Giant Panda-related social media content were more likely to evaluate the Base’s practices positively, highlighting the growing role of digital technologies in shaping visitor attitudes.

These findings are broadly consistent with prior work that emphasizes the effectiveness of Panda Base in delivering conservation and welfare outcomes [48]. However, our results add nuance by identifying social media as a distinctive factor that mediates perceptions of governance and welfare, echoing recent studies on the intersection of technology, animals, and human society [57,58,59,60].

Beyond these immediate findings, our analysis highlights deeper structural challenges in wildlife tourism. A key question arises: are tourists’ positive perceptions of Panda Base genuine evaluations of its governance and welfare practices, or are they shaped by the structural logics of zoos and animal care institutions? Rutherford [32] would suggest the latter. He argues that institutions such as natural history museums deploy “science lite” narratives that simplify life and encourage visitors to consume ordered and measurable truths about nature. Panda Base, as a self-identified scientific research institution, similarly exerts authority through science. While this framing fosters public trust and respect for its conservation mission, it also narrows the space for critical reflection and independent evaluation. Structurally, such dynamics align with broader critiques of nature-based tourism as a hybrid of education and consumerism [22,61], in which visitors derive satisfaction from feeling they are contributing to conservation while simultaneously participating in commodified experiences. In this light, the public’s positive perceptions of Panda Base may be less a reflection of independent judgment and more an outcome of institutionalized and commodified modes of experience.

This raises a critical question for wildlife tourism research: can tourists be considered qualified evaluators of welfare, governance, and conservation performance at WAT destinations such as the Panda Base? How might wildlife tourism give voice to, and empower, tourists in these processes? On the one hand, tourist perceptions matter because they shape public legitimacy, funding, and ongoing support for institutions, as one of the most significant—arguably the most significant—stakeholders in sustaining the wildlife tourism industry, so tourists’ opinions cannot easily be dismissed. Moreover, tourists have direct experiential encounters with governance practices, welfare conditions, and conservation messaging at wildlife venues, which suggests that their perspectives hold intrinsic value [62,63,64]. As Cole [65] argues, sustainable tourism begins with transforming participation from a passive or rhetorical exercise into genuine empowerment. From this perspective, envisioning better-governed WAT venues with stronger animal welfare and conservation practices may begin not only with institutional reforms and scientific inputs but also with enabling tourists to play a more active and critical role.

On the other hand, if those perceptions are heavily mediated by institutional framing, affective attachment to charismatic species, and consumerist logic, their validity as independent assessments of animal welfare or governance standards should be critically examined. Rather than treating visitor perceptions as transparent indicators of WAT performance, scholars and practitioners may need to approach them as socially and structurally conditioned reflections [32,66]. These insights are valuable not for their technical accuracy, but precisely for what they reveal about the dynamics of trust, authority, and commodification in wildlife tourism. The challenge, then, is not to dismiss tourist evaluations but to develop methods and tools that empower visitors to provide more informed, critical, and constructive contributions to governance, welfare, and conservation agendas.

Our results on social media engagement provide one possible avenue for such empowerment see also [57,67]. Tourists with prior experience with Giant Pandas on social media reported more favourable evaluations of Panda Base’s governance, welfare, and conservation than those without such exposure. While this difference highlights how mediated encounters can shape perceptions, it also suggests that information channels—such as digital storytelling, conservation campaigns, or interactive education via social platforms—can influence how visitors interpret and evaluate their experiences [58]. In this sense, social media demonstrates that tourist evaluations are not fixed but can be shaped by the circulation of knowledge and values beyond the site itself [11]. With careful design, such channels could foster more critical, informed, and reflexive forms of visitor assessment. Rather than reducing tourists to passive consumers of institutional narratives, social media and other participatory tools might enable them to act as more knowledgeable stakeholders in advancing welfare, governance, and conservation agendas at WAT destinations.

## 6. Limitations, Implications and Future Directions

While this study provides valuable insights into tourist perceptions of governance, conservation, and animal welfare at the Panda Base, several limitations must be acknowledged. First, the study relies on self-reported perceptions, which may be influenced by social desirability bias or affective attachment to Giant Pandas, potentially inflating positive evaluations. Second, the survey captures a snapshot in time at a single site, limiting generalizability to other wildlife tourism destinations and to longitudinal trends. Third, although our findings highlight social media as an influential factor, we did not directly measure the quality or content of these mediated experiences, which may vary widely and shape visitor perceptions differently.

Despite these limitations, the findings carry important practical and policy implications. Tourist perceptions, although potentially shaped by institutional framing, remain foundational for “using tourism as a conservation tool” [68]. Panda Base and similar venues could capitalize on this by co-designing participatory evaluation tools that enable visitors to provide more informed, critical, and constructive feedback on welfare, governance, and conservation practices, both online and offline. Furthermore, social media, digital storytelling, and interactive education platforms offer promising avenues to empower tourists, providing opportunities for lifelong learning that transcend demographic differences. In this regard, online platforms appear particularly well suited to facilitate what Dierking and Falk [69] describe as “free-choice learning,” allowing visitors to engage voluntarily, reflectively, and meaningfully with conservation and welfare knowledge.

Future research should extend these findings in several directions. Comparative studies across multiple wildlife tourism sites could reveal whether patterns observed at Panda Base are unique or are consistent globally. Longitudinal designs help assess whether visitor perceptions change over time or with repeated exposure. Integrating qualitative approaches, such as interviews or digital ethnography, could deepen understanding of how tourists interpret and evaluate animal welfare and governance. Finally, mixed-methods research combining visitor perceptions with objective welfare audits would provide a more comprehensive evaluation of WAT performance, balancing tourists’ experiential insights with expert assessments.

By addressing these avenues, researchers and practitioners can enhance both the scientific rigour of wildlife tourism studies and visitors’ participatory roles in advancing animal welfare, governance, and conservation agendas.

## Figures and Tables

**Table 1:** An example of the original texts and translation.

Original Texts in the Work of Fennell et al. [49]	Chinese
I1. Locus of power and decision-making in protecting the interests of animals	I1. 保护动物利益的权利和决策中心
a. Governance through cooperative management involving several stakeholders, including citizens, industry, NGOs and the state	a. 基地通过合作管理进行决策，涉及公民、工业、非政府组织和国家等多方利益相关者。
b. Industry and state only	b. 基地仅涉及行业以及国家决策
c. Venue only/Information unavailable	c. 仅限场地/无法获得信息
d. I am unaware of evidence of this criterion at Panda Base	d. 我对熊猫基地是否存在这方面信息不了解

**Table 2:** Demographic characteristics and visiting experiences of participants.

Measure	*n*	%
**Age**	1092	Mean = 31.50; Median = 29.00; SD = 10.223
**Gender**		
Female	671	61.4
Male	381	34.9
I prefer not to tell	40	3.7
**Level of education**		
Primary school and less	18	1.6
High school	98	9.0
Some college and less	291	26.6
Bachelor	559	51.2
Postgraduate and above	126	11.5
**Marital status**		
Single/Divorced	532	48.7
Married	560	51.3
Your place of residence * in the past three years:		
First-tier region	203	18.6
Second-tier region	217	19.9
Chengdu	136	12.5
Third-tier region	536	49.1
**Occupation**		
Full-time	664	60.8
Student	137	12.5
Freelance/Part-time	185	16.9
Retired	43	3.9
Househusband/wife	63	5.8
**This is my first visit to Panda Base**		
Yes	921	84.3
No	171	15.7
**Do you consider yourself a panda fan? (5-Likert scale: 1 = Very unlikely; 2 = Unlikely; 3 = Neutral; 4 = Likely; 5 = Absolutely**	1092	Mean = 4.28; Median = 5.00; SD = 0.894
**Have you followed a panda-themed social media account?**		
Yes	851	77.9
No	241	22.1

Note: * This classification of Chinese cities is based on commonly recognized administrative and economic hierarchies. First-tier cities refer to the largest and most economically developed urban centers, such as Beijing, Shanghai, Guangzhou, and Shenzhen. Second-tier cities typically include provincial capitals and other major regional hubs with strong economic performance and cultural influence. Third-tier cities generally encompass smaller prefecture-level municipalities with more limited economic and infrastructural development. Chengdu, while usually considered a second-tier city, is treated separately in this study because previous research by the second author has shown that local residents from Chengdu constitute a disproportionately dominant share of visitors to the Panda Base.

**Table 3:** Participants’ Perception of Governance, Welfare, and Conservation.

		Mean	SD	Variance	Skewness	Kurtosis	Percentiles		
							25	50	75
Governance	I1	1.63	0.982	0.964	1.490	0.994	1.00	1.00	2.00
	I2	1.56	0.900	0.809	1.679	1.880	1.00	1.00	2.00
	I3	1.60	0.940	0.884	1.599	1.475	1.00	1.00	2.00
	I4	1.53	0.900	0.810	1.783	2.202	1.00	1.00	2.00
	I5	1.61	0.951	0.904	1.589	1.425	1.00	1.00	2.00
	I6	1.66	0.953	0.908	1.446	1.039	1.00	1.00	2.00
	I7	1.57	0.921	0.849	1.690	1.827	1.00	1.00	2.00
	I8	1.56	0.893	0.797	1.705	2.018	1.00	1.00	2.00
	I9	1.60	0.932	0.868	1.612	1.600	1.00	1.00	2.00
Conservation	II1	1.47	0.936	0.876	1.973	2.519	1.00	1.00	2.00
	II2	1.46	0.869	0.755	2.007	3.038	1.00	1.00	2.00
	II3	1.40	0.858	0.735	2.240	3.883	1.00	1.00	1.00
	II4	1.92	1.291	1.668	0.875	−1.078	1.00	1.00	4.00
Welfare	III1	1.47	0.923	0.852	1.914	2.325	1.00	1.00	2.00
	III2	1.50	0.932	0.868	1.822	2.043	1.00	1.00	2.00
	III3	1.50	0.983	0.967	1.852	1.929	1.00	1.00	2.00
	III4	1.50	0.935	0.874	1.825	2.034	1.00	1.00	2.00
	III5	1.55	0.937	0.878	1.627	1.437	1.00	1.00	2.00
	III6	1.40	0.901	0.812	2.148	3.141	1.00	1.00	1.00
	III7	1.45	0.948	0.899	1.967	2.360	1.00	1.00	1.00
	III8	1.39	0.901	0.811	2.213	3.386	1.00	1.00	1.00
	III9	1.38	0.887	0.786	2.269	3.718	1.00	1.00	1.00
	III10	1.64	1.111	1.235	1.407	0.278	1.00	1.00	2.00
	III11	1.47	0.978	0.957	1.890	1.997	1.00	1.00	1.00
	III12	1.48	1.004	1.009	1.869	1.822	1.00	1.00	1.00

**Table 4:** Two-category demographic characteristics tested for *t*-test.

	Variables	Demographic Categories			*t*	*p*
		Group 1	Group 2	Difference		
Marital status		Single/Divorced	Married			
	I2	1.48 (0.872)	1.61 (0.903)	0.15	2.306	0.021
	I4	1.46 (0.848)	1.59 (0.933)	0.13	2.478	0.013
	I7	1.48 (0.881)	1.63 (0.945)	0.15	2.633	0.009
	I8	1.49 (0.853)	1.61 (0.920)	0.12	2.276	0.023
	II4	2.06 (1.344)	1.82 (1.238)	0.24	3.013	0.003
	III2	1.43 (0.863)	1.55 (0.974)	0.12	2.070	0.039
	III6	1.32 (0.784)	1.46 (0.971)	0.14	2.682	0.007
		Female	Male			
Gender	III6	1.42 (0.915)	1.47 (0.936)	0.05	2.218	0.027
	III9	1.31 (0.820)	1.49 (0.959)	0.18	3.101	0.002
Visitation frequency		First-time	Repeat			
	I6	1.62 (0.920)	1.84 (1.099)	0.22	2.384	0.018
	I7	1.54 (0.894)	1.72 (1.047)	0.18	2.131	0.034
	I9	1.58 (0.912)	1.75 (1.024)	0.17	2.038	0.043
	II4	1.86 (1.268)	2.27 (1.364)	0.41	3.713	0.000
	III2	1.47 (0.910)	1.65 (1.032)	0.18	2.095	0.037
	III10	1.59 (1.076)	1.92 (1.251)	0.33	3.278	0.001

Note. Standard deviations are shown in parentheses. The table reports only values where *p* < 0.05. Response options are coded as follows: Choice A = 1, Choice B = 2, Choice C = 3, and Choice D = 4.

**Table 5:** Kruskal–Wallis results on educational backgrounds.

		Education				Kruskal–Wallis H (df = 3)	*p*
		High School and Less (*n* = 116)	Some College (*n* = 291)	Bachelor (*n* = 559)	Postgraduate and Above (*n* = 126)		
Governance	I1	1.78 (1.148)	1.68 (1.016)	1.58 (0.906)	1.65 (1.046)	1.912	0.591
	I2	1.66 (1.014)	1.57 (0.908)	1.52 (0.840)	1.62 (1.019)	0.699	0.873
	I3	1.69 (1.058)	1.61 (0.953)	1.55 (0.896)	1.66 (0.989)	1.340	0.720
	I4	1.62 (0.993)	1.60 (0.936)	1.47 (0.829)	1.60 (1.013)	4.660	0.198
	I5	1.78 (1.096)	1.61 (0.923)	1.55 (0.904)	1.70 (1.053)	4.464	0.215
	I6	1.74 (0.988)	1.66 (0.964)	1.64 (0.938)	1.63 (0.968)	1.407	0.704
	I7	1.73 (1.033)	1.55 (0.921)	1.53 (0.875)	1.61 (1.004)	3.888	0.274
	I8	1.70 (0.998)	1.58 (0.919)	1.51 (0.829)	1.58 (0.991)	3.502	0.320
	I9	1.75 (1.046)	1.64 (0.931)	1.54 (0.887)	1.67 (1.003)	5.642	0.130
Conservation	II1	1.64 (1.091)	1.45 (0.917)	1.42 (0.892)	1.56 (1.000)	5.664	0.129
	II2	1.67 (1.070)	1.48 (0.888)	1.41 (0.822)	1.44 (0.795)	6.083	0.108
	II3	1.63 (1.092)	1.42 (0.869)	1.35 (0.790)	1.39 (0.848)	6.422	0.093
	II4	1.71 (1.187)	1.83 (1.225)	1.96 (1.308)	2.17 (1.415)	7.796	0.050
Welfare	III1	1.62 (1.060)	1.46 (0.929)	1.43 (0.895)	1.49 (0.892)	4.167	0.244
	III2	1.66 (1.079)	1.49 (0.930)	1.45 (0.891)	1.58 (0.958)	5.551	0.136
	III3	1.68 (1.116)	1.46 (0.969)	1.45 (0.928)	1.66 (1.097)	10.206	0.017
	III4	1.70 (1.081)	1.48 (0.930)	1.47 (0.917)	1.50 (0.865)	6.630	0.085
	III5	1.66 (1.103)	1.51 (0.904)	1.53 (0.919)	1.65 (0.924)	5.048	0.168
	III6	1.66 (1.104)	1.40 (0.882)	1.37 (0.876)	1.31 (0.815)	12.369	0.006
	III7	1.70 (1.097)	1.43 (0.927)	1.40 (0.912)	1.50 (0.978)	12.139	0.007
	III8	1.61 (1.102)	1.44 (0.954)	1.33 (0.826)	1.32 (0.855)	10.902	0.012
	III9	1.54 (1.033)	1.37 (0.878)	1.37 (0.875)	1.31 (0.805)	5.499	0.139
	III10	1.78 (1.127)	1.59 (1.102)	1.59 (1.085)	1.84 (1.209)	11.733	0.008
	III11	1.71 (1.135)	1.48 (1.011)	1.40 (0.904)	1.56 (1.031)	11.946	0.008
	III12	1.57 (1.057)	1.53 (1.048)	1.42 (0.968)	1.52 (1.010)	6.221	0.101

Note. Standard deviations are shown in parentheses. The table reports only values where *p* < 0.05. Response options are coded as follows: Choice A = 1, Choice B = 2, Choice C = 3, and Choice D = 4.

**Table 6:** *t*-test for visitors with or without social media engagement.

		Have You Followed Giant Panda-Related Topics on Social Media?			*t*	*p*
		*Yes*	*No*	Difference		
Governance	I1	1.55 (0.926)	1.92 (1.115)	0.37	4.630	0.000
	I2	1.50 (0.852)	1.76 (1.028)	0.26	3.617	0.000
	I3	1.52 (0.875)	1.88 (1.098)	0.36	4.734	0.000
	I4	1.48 (0.843)	1.73 (1.055)	0.25	3.501	0.001
	I5	1.53 (0.888)	1.90 (1.099)	0.37	4.870	0.000
	I6	1.57 (0.891)	1.95 (1.096)	0.38	4.966	0.000
	I7	1.49 (0.852)	1.85 (1.089)	0.36	4.808	0.000
	I8	1.51 (0.855)	1.72 (1.001)	0.21	3.011	0.003
	I9	1.54 (0.879)	1.83 (1.069)	0.29	3.850	0.000
Conservation	II1	1.38 (0.849)	1.76 (1.148)	0.38	4.736	0.000
	II2	1.41 (0.816)	1.63 (1.017)	0.22	3.005	0.003
	II3	1.35 (0.793)	1.59 (1.033)	0.24	3.432	0.001
	II4	1.82 (1.243)	2.29 (1.391)	0.47	4.806	0.000
Welfare	III1	1.42 (0.868)	1.64 (1.079)	0.22	2.989	0.003
	III2	1.45 (0.871)	1.68 (1.103)	0.23	3.074	0.002
	III3	1.42 (0.896)	1.80 (1.199)	0.38	4.552	0.000
	III4	1.43 (0.851)	1.73 (1.157)	0.30	3.706	0.000
	III5	1.50 (0.882)	1.74 (1.093)	0.24	3.107	0.002
	III6	1.35 (0.839)	1.57 (1.078)	0.22	2.959	0.003
	III7	1.39 (0.873)	1.68 (1.149)	0.29	3.615	0.000
	III8	1.32 (0.813)	1.62 (1.131)	0.30	3.799	0.000
	III9	1.31 (0.801)	1.63 (1.107)	0.32	4.163	0.000
	III10	1.57 (1.055)	1.90 (1.258)	0.33	3.738	0.000
	III11	1.41 (0.900)	1.72 (1.185)	0.31	3.796	0.000
	III12	1.38 (0.903)	1.81 (1.246)	0.43	4.987	0.000

Note. Standard deviations are shown in parentheses. The table reports only values where *p* < 0.05. Response options are coded as follows: Choice A = 1, Choice B = 2, Choice C = 3, and Choice D = 4.

## Data Availability

The original contributions presented in this study are included in the article. Further inquiries can be directed at the corresponding authors.

## Appendix A

Governance, Conservation, and Welfare
Research project
Principle Investigator
Name: Yulei Guo
Affiliation: Chengdu Research Base of Giant Panda Breeding
Phone: 14726198960
Email: yulei.guo@panda.org.cn
Research aim
We kindly invite you to participate in a scientific survey. Before you decide to take part in this study, please ensure that you understand the purpose of this research and the conditions it will involve. Please read the following information carefully. If you have any questions or need more information, please consult the researcher.
This study aims to assess Panda Base’s performance in governance, conservation, and animal welfare.
Research process
This study is an online survey. In the survey, you will be required to provide related demographic information. Also, you need to answer questions about your preference for animals at Panda Base.
The survey will take about 5 min.
Research risk
There are no known risks associated with completing this survey. You may choose to decline to answer some or all of the questions. If you wish, you can terminate your questionnaire at any time.
Benefits of research
Your answer will help us better evaluate the performance of Panda Base and improve visitor satisfaction.
Privacy
Your responses to this questionnaire will be treated confidentially. Please do not disclose any personal identification information in the questionnaire.
Contact info
If you have any questions about this study or if you experience any adverse effects as a result of participating in this research, you may contact the researchers. The researchers’ contact information has been provided in the previous text. If you have any concerns about your rights as a research participant, please contact Yulei Guo.
Voluntary participation
You are participating in this study voluntarily. Whether you participate in this study is entirely your own decision. If you choose to participate in this research, you will be asked to sign an informed consent form. Even after signing the consent form, you may still withdraw from the study at any time without giving a reason, and this will not affect your relationship with the researchers.
Informed consent form
I have read and understood the provided information and have had the opportunity to ask questions. I am participating in this survey voluntarily and can withdraw at any time without giving a reason.
If you agree and accept the above terms, please click the ‘Agree’ button below to begin answering the survey questions. If you do not agree or accept the above terms, please click the ‘Disagree’ button to terminate this survey.
Do you agree to sign the informed consent form: *

○Yes
○No

Your gender: *

○Male
○Female
○I do not want to tell

Your age: *
_________________________________
Your place of residence in the last 3 years: *
_________________________________
Your education: *

○Primary school
○High School
○Some college and less
○Bachelor
○Postgraduate and above

Marital status: *

○Single
○Married
○Devoiced

Your current occupation *

○Full-time employment	○Student
○Househusband/wife	○Freelance
○Retired	

Have you encountered giant pandas already? *

○Yes
○No

This is my first visit to Panda Base *

○Yes
○No

Have you engaged with panda-themed social media content? *

○Yes
○No

Governance at Panda Base (Online & Offline)
* I1. Locus of power and decision-making in protecting the interest of animals

○Governance through cooperative management involving several stakeholders, including citizens, industry, NGOs and the state.
○Industry and state only.
○Venue only.
○I am unaware of evidence of this criterion at Panda Base.

* I2. Application of Corporate Social Responsibility (CSR) & sustainability values for best practice

○CSR and sustainability are central to Panda Base’s operations.
○CSR and sustainability are a minor focus of operations.
○Complete lack of SCR and sustainability in operations.
○I am unaware of evidence of this criterion at Panda Base.

* I3. Reporting and communication of operations (e.g., annual report made publicly available)

○Extensive reporting and communication of operations made easily available.
○Limited reporting and communication of operations and/or not easily available;
○No reporting and communication of operations;
○I am unaware of evidence of this criterion at Panda Base

* I4. Truth in marketing (e.g., Internet claims versus experience)

○Products conform to marketing claims;
○No conformity to marketing and education claims;
○Deliberate greenwashing;
○I am unaware of evidence of this criterion at Panda Base

* I5. Provision of economic benefits to local people from the venue (e.g., jobs)

○Active role (and policy) around providing jobs for local people;
○Jobs provided, but no deliberate policy;
○No effort to provide economic benefits to local people;
○I am unaware of evidence of this criterion at Panda Base

* I6. Pursuit of best practice in the training of employees in welfare and conservation

○Policy and proven evidence of training/skill development for local communities;
○Basic training and allocation of resources, but not in the pursuit of best practices;
○No training of local people in proper welfare and conservation of animals;
○I am unaware of evidence of this criterion at Panda Base

* I7. Role in inducing local communities to support the conservation and welfare of animals, supported by details of how

○Active role inducing communities to support conservation and welfare, regular interaction with the local community;
○No policy, but occasional or one-off efforts to induce the community to support conservation and welfare;
○No connection whatsoever with the community over conservation and welfare support;
○I am unaware of evidence of this criterion at Panda Base

* I8. Support for conservation—or welfare-relevant volunteer programs

○Volunteer programs to support genuine conservation and welfare efforts;
○Volunteer programs to support venue enterprises with no linked welfare or conservation outputs
○No volunteer programs;
○I am unaware of evidence of this criterion at Panda Base

* I9. Regulation of the venue

○Regulation by external authority (e.g., third-party certification schemes) or industry self-regulation;
○Regulation through norms or codes of ethics;
○No regulation;
○I am unaware of evidence of this criterion at Panda Base

Conservation
* II1. Conservation education messages in the tourist experience

○Active participation in appropriate and responsibly managed conservation outcome (e.g., collecting data through citizen science);
○Received conservation education through lecture/program;
○No conservation education or message received;
○I am unaware of evidence of this criterion at Panda Base;

* II2. Tourist uses of animals that impact conservation status;

○Totally benign uses only (e.g., panda viewing);
○Indirect consumption of animals through the purchase of animal products at the venue (souvenirs, eating animals from the venue);
○Direct consumptive practices (e.g., hunting);
○I am unaware of evidence of this criterion at Panda Base

* II3. Conservation benefits of captivity

○Yes, direct conservation benefits such as rehabilitation, restoration, reintroduction, and/or lifetime care;
○Yes, but indirect conservation benefits through education, where evidence shows that education improves tourists’ knowledge of conservation issues and encourages appropriate engagement.
○No conservation benefits;
○I am unaware of evidence of this criterion at Panda Base.

* II4. Stated and validated percentage of income donated towards habitat protection or science/research:

○6% or more
○1–5%
○0%
○I am unaware of evidence of this criterion at Panda Base

Animal Welfare
* III1. Mobility (size and obstructions)

○Animals can explore the large sanctuary environment.
○Some constraints to the exploration of the captive environment;
○Significant constraints to exploration of captive environment (example chains/small cages);
○I am unaware of evidence of this criterion at Panda Base

* III2. Naturalness of captive environment

○Enclosures with characteristics that replicate natural environment, with a low density of individuals per unit area;
○Enclosures contain some characteristics that replicate natural environment, with a higher density of animals per unit area;
○Enclosures lack characteristics that replicate the natural environment and/or appear to have too many individuals per unit area.
○I am unaware of evidence of this criterion at Panda Base

* III3. Enrichment devices/stimuli

○Animals regularly provided with enrichment devices for cognitive, emotional and physical health;
○Animals are periodically given enrichment devices for cognitive, emotional, or physical health.
○No enrichment devices at all;
○I am unaware of evidence of this criterion at Panda Base

* III4. Hygiene

○Animals’ food and environment appear to be of optimal hygiene.
○Animals’ food and environment are moderately hygienic (e.g., excrement not regularly picked up);
○Animals’ food and environment are not at all hygienic (e.g., animal covered in excrement);
○I am unaware of evidence of this criterion at Panda Base

* III5. Noise quality (including tourist noise)

○Little to no noise that may cause disturbance;
○Periodic noise that may cause minor disturbance;
○Sustained noise that may cause major disturbance;
○I am unaware of evidence of this criterion at Panda Base

* III6. Signs of obesity or malnutrition

○Animals appear healthy;
○Minor signs of obesity or malnutrition;
○Outward signs of obesity or malnutrition;
○I am unaware of evidence of this criterion at Panda Base

* III7. Nutrition

○Evidence from venue that they feed animals a diet that replicates food from their natural environment.
○Animals fed a combination of natural food and non-natural treats;
○Animals fed predominantly non-natural, high-calorie treats;
○I am unaware of evidence of this criterion at Panda Base

* III8. Entertainment intensity

○Animals free from having to entertain tourists (Not handled for educational purposes or made to do shows);
○Animals used for educational purposes (e.g., demonstrating to tourists for limited periods);
○Animals are made to perform (jumping through burning hoops, etc.) regularly;
○I am unaware of evidence of this criterion at Panda Base

* III9. Touch policy for tourists (e.g., selfies, cut petting, feeding).

○No touching (e.g., petting, handfeeding, or selfies allowed;
○Time-limited petting or selfies allowed, with evidence of strict policing;
○Petting, feeding, and or selfies allowed;
○I am unaware of evidence of this criterion at Panda Base

* III10. Reinforcement

○Animals regularly receive positive reinforcement;
○Animals receive both positive and negative reinforcement;
○Animals receive only negative reinforcement;
○I am unaware of evidence of this criterion at Panda Base

* III11. Abuse-respect continuum

○Never any abuse; abundant care and respect;
○Some care and respect in the absence of abuse;
○Evidence of outward abuse, pain and suffering;
○I am unaware of evidence of this criterion at Panda Base

* III12. Visitor control measures

○Evidence of regular control of visitor numbers, group sizes, and group behaviour;
○Evidence of periodic control of visitor numbers, group sizes, and group behaviour;
○Evidence of no visitor control of numbers, group sizes, and group behaviour;
○I am unaware of evidence of this criterion at Panda Base
